# Ultrasonographic Diagnosis of an Os Styloideum in an Adolescent Volleyball Player

**DOI:** 10.7759/cureus.31573

**Published:** 2022-11-16

**Authors:** Hector M Rivera-Melo

**Affiliations:** 1 Diagnostic Imaging, Southern California University of Health Sciences, Whittier, USA

**Keywords:** musculoskeletal radiology, pediatric sports medicine, wrist, diagnostic musculoskeletal ultrasound, os styloideum

## Abstract

The os styloideum is an uncommon accessory ossicle located at the dorsum of the wrist, which may present with symptoms similar to those of a ganglion cyst. The presumed etiology of the os styloideum is congenital non-fusion of cartilaginous components about the carpals. Various imaging modalities, including ultrasonography, can be used to differentiate this entity from various other sources of painful dorsal-sided wrist nodules.

A 13-year-old female presented with dorsal wrist pain and focal swelling. She reported that the symptoms began two months prior to the initial evaluation. A clinical diagnosis of a ganglion cyst was made. The initial radiographic examination was interpreted as negative. Sonographic examination revealed the presence of an accessory ossicle consistent with an os styloideum, which was later confirmed on the initial radiographic study.

The patient elected for a trial of conservative treatment, which included activity modification and functional taping. This resulted in moderate, but not complete, reduction in pain and improvement in function.

The clinical presentation of a painful dorsal nodule about the wrist should prompt the consideration of an os styloideum. This case demonstrates the use of diagnostic ultrasound as a modality for evaluating and confirming the diagnosis of an os styloideum.

## Introduction

The os styloideum is an accessory ossicle of the wrist located at the carpometacarpal junction dorsally in approximately 0.3%-2.3% of wrists [1]. Amongst all accessory ossicles of the wrist, the os styloideum is the most likely to present with symptoms [2]. Imaging findings associated with a painful os styloideum include bone marrow edema and partial fusion with adjacent osseous structures as seen on MRI [3]. The general term for a bony prominence at the dorsum of the wrist is also sometimes referred to as a ‘carpal boss’. The carpal boss is an entity with numerous implicated etiologies [4]. Congenital non-fusion of developing cartilaginous components is a commonly cited mechanism for the development of an os styloideum [4]. Traumatic and arthritic origins have also been suggested as sources for the carpal boss [1,4]. The clinical presentation of an os styloideum may closely simulate the clinical presentation of a dorsal ganglion cyst of the wrist [3]. The following case presents with clinical features shared by a carpal boss and a ganglion cyst.

## Case presentation

A 13-year-old, right-hand-dominant, female volleyball player presented with right-sided dorsal wrist pain and focal swelling. She described moderate pain and pressure that occasionally traveled distally to her right metacarpophalangeal joints and proximally to her right forearm. She initially noticed a mass on her wrist two months prior to presentation, at which time she began to feel pain while performing maneuvers in practice that involve terminal wrist extension such as setting and hitting. The patient also reported an increase in pain while writing in class. The pain and amount of swelling reportedly varied throughout the course of a day and the patient stated that resting her wrist was palliative. The initial working diagnosis was a dorsal ganglion cyst of the wrist.

Physical examination of the wrist demonstrated a small, firm palpable mass on the dorsum of the right wrist, which she rated a 4/10 on the numeric pain rating scale. The mass itself was graded as +2 tenderness to palpation and the wrist extensor tendons were graded as +1 tenderness. No discoloration of the area in question was noted. The patient had moderately limited and painful radiocarpal extension. Manual muscle testing revealed full strength (5/5) in both wrists, though resisted wrist and digit extension reproduced her pain on the side of the mass. Special tests as part of a wrist screening protocol including Scaphoid shift and Phalen’s test were performed with negative results.

**Figure 1:**
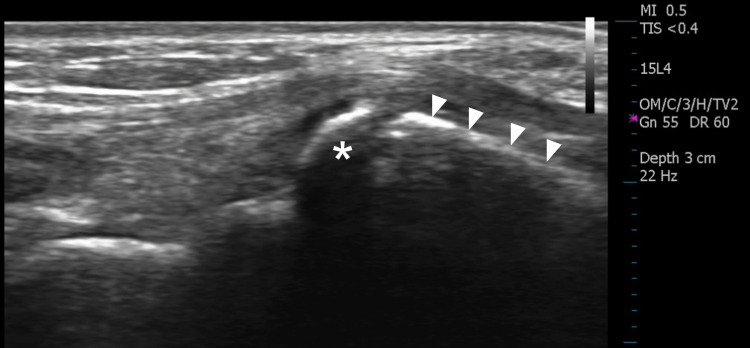
Longitudinal gray scale ultrasound view of the base of the symptomatic third metacarpal (arrowheads) demonstrating an accessory ossicle (asterisk). The left side of the image is proximal.

**Figure 2:**
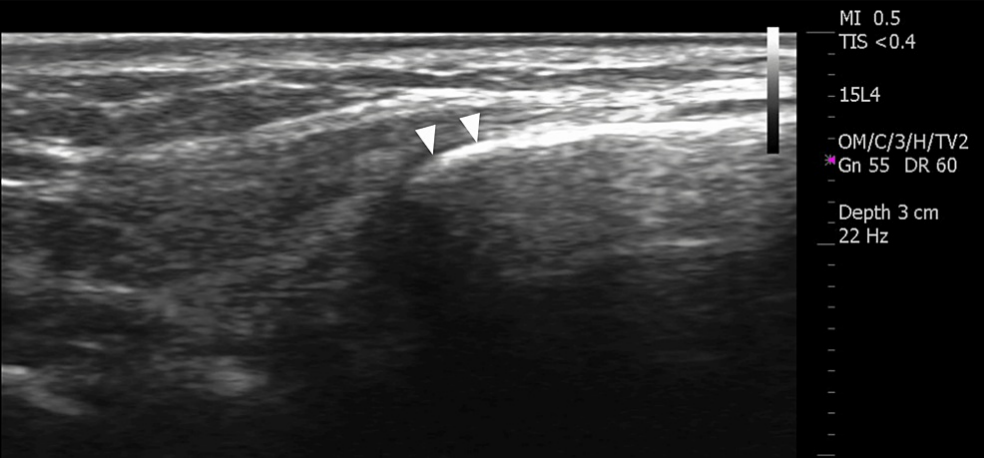
Longitudinal gray scale ultrasound view of the base of the asymptomatic, contralateral third metacarpal (arrowheads) for comparison. The left side of the image is proximal.

**Figure 3:**
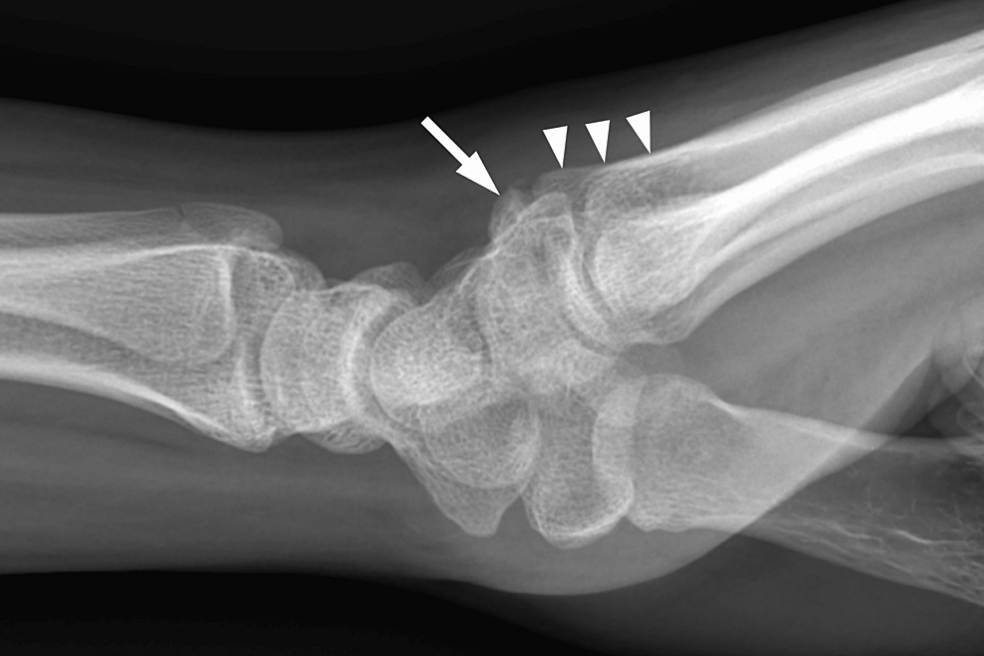
Lateral radiographic projection of the symptomatic wrist demonstrating a small ossicle (arrow) adjacent to the base of the third metacarpal (arrowheads).

A three-view radiographic study of the wrist was ordered, which was initially interpreted as normal. Six days after the radiographs were obtained, a diagnostic ultrasound examination of the right dorsal wrist, including short- and long-axis views of the area in question was performed using a 15 MHz linear transducer. The sonographic examination demonstrated a hyperechoic osseous prominence that formed an irregular junction with the base of the third metacarpal (Figure 1). Doppler evaluation of the region was negative for hyperemia. The asymptomatic (left) wrist was scanned for comparison and demonstrated a normal base of the third metacarpal without an associated osseous mass (Figure 2). Upon re-evaluation of the initial radiographic exam, the lateral projection demonstrated a well-corticated ossicle adjacent to the base of the third metacarpal consistent with the diagnosis of an os styloideum (Figure 3).

The patient opted for a conservative course of treatment, which included manual release therapy of the wrist extensor muscles and functional taping of the wrist during sports with elastic tape placed transversely across the dorsum of the proximal carpal row intended to provide a mild compressive/stabilizing effect of the wrist, while allowing for a full active range of motion in all directions. Recommendations also included short-term activity modification while playing volleyball to reduce maneuvers such as setting and hitting that require the most wrist extension. Concordantly, the patient began playing in the "middle blocker" and "libero" positions more frequently, which facilitated this recommendation. The patient reported a reduction in pain level to a 2/10 on the numeric pain rating scale three weeks after conservative measures were implemented, which was tolerable enough for her to return to play. This pain level persisted for a period of six months while performing wrist extension motions, and the patient was pain-free at rest.

## Discussion

The os styloideum is an accessory ossicle, which develops during embryogenesis and is most commonly encountered at the junction between the second metacarpal base, the third metacarpal base, the capitate, and trapezoid bones [1,5,6]. These ossicles may be partially united to the adjacent osseous structures or may be separated by a fibrous synchondrosis [3]. Although the typical age for presentation of an os styloideum is in the fourth decade, there are several reported cases in the literature of ossicles encountered in the pediatric population [4,7]. An increased incidence of os styloideum has been reported among professional hockey players and boxers; however, an association amongst volleyball players has yet to be documented in the current literature [7,8].

Clinically speaking, many cases of os styloideum are asymptomatic and may be diagnosed incidentally [4]. Diagnosis of an os styloideum can be made via conventional radiographs, although CT, MRI, and ultrasound have been reported as useful [1-4,6]. MRI has demonstrated the presence of marrow edema in some symptomatic cases [3]. Ultrasound is considered a useful modality for quickly differentiating a superficial nodule as fluid-filled or osseous [6]. Most fluid-filled cysts such as ganglia appear hypoechoic or anechoic on ultrasound with characteristic through transmission enhancement and occasional internal septations. In contrast, the outer cortical surface of an accessory ossicle will appear hyperechoic and demonstrate acoustic shadowing deep to the structure. In the case of an os styloideum, an accessory articulation may be appreciated on ultrasound between the base of the second and/or third metacarpal and the distal aspect of the ossicle, as depicted in this case. Two essential elements in differentiating accessory ossicles from acute fractures about the wrist are the well-corticated margins of an ossicle and the familiarity with the locations of common ossicles such as the os styloideum [1,2]. Other common causes of painful nodules at the dorsal wrist include ganglion cyst formation, tendinopathy, degenerative osteophyte formation, distended bursae, post-traumatic osseous changes, gouty tophi, and benign neoplasms such as giant cell tumor of tendon sheath or lipomas [1,4,5,9].

Treatment methods for a symptomatic os styloideum include local corticosteroid injection, nonsteroidal anti-inflammatory agents, splinting, and surgical resection with variable rates of success and complications reported for each intervention [4,5]. Currently, there is no consensus for a gold standard of management and cases are often treated based on the severity of symptoms [4].

## Conclusions

Occasionally, the presence of an accessory ossicle in the wrist may present with pain and may simulate a ganglion cyst upon clinical presentation. This case highlights how ultrasound imaging can serve an important role in differentiating between the diagnosis of a ganglion cyst and an os styloideum when evaluating a dorsal-sided wrist mass with pain. Conservative treatment of an os styloideum shows various degrees of success and should be trialed before advancing to surgical interventions.

## References

[1] Goiney C, Porrino J, Richardson ML, Mulcahy H, Chew FS (2017). Characterization and epidemiology of the carpal boss utilizing computed tomography. J Wrist Surg.

[2] Hansford BG (2020). Multimodality pitfalls of wrist imaging with a focus on magnetic resonance imaging: what the radiologist needs to know. Top Magn Reson Imaging.

[3] Poh F (2015). Carpal boss in chronic wrist pain and its association with partial osseous coalition and osteoarthritis - a case report with focus on MRI findings. Indian J Radiol Imaging.

[4] Kaniewska M, Haefeli M, Laesser U, Niemann T (2017). That's my STYLEoideum - symptomatic os styloideum in an adolescent male. J Radiol Case Rep.

[5] Karmazyn B, Siddiqui AR (2002). Painful os styloideum in a child. Pediatr Radiol.

[6] Arend CF (2014). The carpal boss: a review of different sonographic findings. Radiol Bras.

[7] Greditzer HG 4th, Hutchinson ID, Geannette CS, Hotchkiss RN, Kelly BT, Potter HG (2017). Prevalence of os styloideum in National Hockey League players. Sports Health.

[8] Park MJ, Namdari S, Weiss AP (2008). The carpal boss: review of diagnosis and treatment. J Hand Surg Am.

[9] Jadhav S, Awasthi A, Deshpande S, Jadawala V, Salwan A (2022). Giant cell tumor of extensor tendon sheath in ring finger: a case report. Cureus.

